# Editorial: Evidence to practice: Bridging the gap in environmental challenges (cold, heat, hypoxia) in sport and exercise: Acclimatization/acclimation, training, competitions, recovery, rehabilitation and therapeutic interventions

**DOI:** 10.3389/fspor.2022.1090086

**Published:** 2022-11-17

**Authors:** Franck Brocherie, Joao Brito, Julio A. Costa, Gregoire P. Millet

**Affiliations:** ^1^Laboratory Sport, Expertise and Performance (EA 7370), French Institute of Sport, Paris, France; ^2^Portugal Football School, Portuguese Football Federation, Lisbon, Portugal; ^3^Faculty of Biology and Medicine, University of Lausanne, Lausanne, Switzerland

**Keywords:** environmental stressors, hypoxic dose, thermoregulation, humid and tropical condition, adaptation

## Our research topic

Aiming to improve the translation of applied sport science findings to evidence-based practice in real-life sport performance environments, Frontiers has launched a series of “Evidence to Practice” Research Topics that bridge the gap, for instance, in injury prevention, sport nutrition or environmental challenges. Based on the scientific interest in this latter field [e.g., (1) for hypoxia], the present Research Topic is dedicated to the influence, benefits and limits of cold, heat and hypoxia (including acclimatization/acclimation) on exercise training and sports competitions, with their potential translation to rehabilitation, prevention and therapeutic protocols.

## New insights

This Research Topic contains seven original researches and one brief research report published either in Frontiers in Physiology (Exercise Physiology; Environmental, Aviation and Space Physiology) or Frontiers in Sports and Active Living (Elite Sports and Performance Enhancement) that focused on the effect of cold (*n* = 2), heat (*n* = 3) or hypoxia (*n* = 3).

### Temperature-related interventions

In Winter endurance sports such as cross-country skiing, exposure to cold during training and competition could lead to hypothermia and/or frostbite, if core temperature (*T*_core_) or skin temperature (*T*_skin_) decline to critical levels (2). To improve safety in cold, the International Olympic Committee called for *in-situ* measure of metabolic heat production rates. Blokker et al. reported the thermal response (i.e., heat flux, *T*_core_ and *T*_skin_ at different body parts) of elite biathletes during a cross-country skiing bout at moderate intensity (i.e., ~14–15 mins at ~75% of maximal heart rate) in a cold environment (i.e., < -2 ± 1°C and < 89 ± 5% relative humidity). The authors observed a large decrease in *T*_skin_ and an increase in heat flux for the thigh, back, anterior and lateral thorax, despite a moderate increase in *T*_core_. Given the fact that a lower *T*_skin_ reduces muscle temperature and activity (3) and subsequently aerobic performance (4), *these findings provide potential guidance for clothing strategy and race suit design for Winter sports*.

Cold exposure has also been used as therapy to reduce post-traumatic edema and inflammation, and to facilitate post-exercise recovery (5). However, whether frequent whole-body cryotherapy interferes with long-term adaptations to concurrent training remains debated. Haq et al. investigated the impact of two sessions per week of 3-min whole-body cryotherapy (30 s at −60°C and 150 s at −120°C) on adaptations during a 6-week program involving concurrent endurance, strength, and power training. Compared to controls, repetitive whole-body cryotherapy did not blunt adaptations and did not negatively impact fitness attributes, except for muscular power. In terms of periodization, *repetitive whole-body cryotherapy enhanced post-exercise recovery, in particular during phases focusing on strength development, but may limit the development of muscular power*.

Exercising in the heat induces a physiological strain that leads to decreased performance over time. In team sports, the application of ~15-min whole-body or lower-limb cold-water immersion during half-time appears unrealistic. Zhang et al. therefore tested whether a short (i.e., 3 min) whole-body or lower-limb cold-water immersion implemented within a 15-min half-time break would have an ergogenic effect on subsequent soccer-specific performance in the heat (wet bulb globe temperature = 39°C). The results indicate that such *short-term whole-body and, in a lower extent, lower-limb cold-water immersion attenuated the impairment of intermittent-running performance* generally observed in match-play second half [e.g., (6)], *through a decrease in T*_skin_ *and improvement of perceived strain*. Inversely, Eimantas et al. examined whether a short-duration (5 min) whole-body hot water immersion (45°C) could increase or impair neurophysiological performance, due to the production of a strong neural and temperature flux without inducing hyperthermia. In young men, *such exposure accelerated calf muscle contractility and electrophysiological modulation/adjustment of motor drive transmission, but without effect on central and peripheral fatigue*.

Finally, whatever the approach used to attenuate the development of hyperthermia, a continuous and real-time monitoring of *T*_core_ is paramount to adequately assess early signs of heat-related disorders. Considering the limitations (i.e., impracticability, inaccuracy, cost) of the actual *T*_core_ monitors, de Korte et al. explored the validity of the estimated-*T*_core_ algorithm that uses sequential heart rate values to predict *T*_core_ in elite athletes exercising in the heat. They found that *the estimated*−*T*_core_ *algorithm should not be used to identify risk of heat*−*related disorders due to low sensitivity and high false*−*negative rate at T*_core_ > *39*°*C*. However, in the low- to mid-range of *T*_core_ values (37.75–38.75°C), its use could be informative during controlled hyperthermia heat acclimation protocols.

### Altitude/hypoxic training

Given the additional stress of hypoxia, altitude training requires careful individual monitoring (7) to optimize acclimatization and performance enhancement. Blood markers, particularly total hemoglobin mass measurement (8), are primary indicators but are invasive, time-consuming and expensive. Karlsson et al. monitored the daily variations in selected subjective and objective variables during a 17–21-day altitude training camp at 1,800 m in elite cross-country skiers and biathletes. They reported that *resting pulse oxygen saturation and resting heart rate did not change systematically throughout the altitude training camp*. This warrants further research on the relevance of (in)direct markers to altitude in elite athletes in reference to planning and periodization, training intensity or recovery.

Nutrition is another key parameter that may influence adaptations to altitude training. Sousa et al. investigated the effectiveness of 4 weeks of high-intensity interval training in normobaric hypoxia with chronic dietary nitrate (${\text{NO}}_{3}^{-}$) supplementation. Based on the results, the authors suggested that *chronic dietary* ${NO}_{3}^{-}$ *supplementation did not bring any additional benefit in such hypoxic high-intensity intermittent training*. Noteworthy, the ability to recover oxygen saturation was faster compared to control. Interestingly, Ma et al. examined the role of silent information regulator 2 homolog 3 (Sirt3)—a mitochondrial deacetylase that affects skeletal muscle microcirculation—in the process of hypoxic training. Six weeks of *hypoxic training in C57BL/6 mice increased Sirt3 levels in skeletal muscle, thereby enhancing skeletal muscle microcirculation function and increasing microcirculatory vasodilation capacity and capillary formation*. This may play a role in skeletal muscle angiogenesis as previously reported in hypoxic training-related human studies (9).

## Concluding remark: Facilitating the evidence-based practice

The integrated combination between research and practice is paramount to improve the quality of any environmental intervention. We recognize that enabling and implementing evidence-based practices is likely a dilemma as there is no ‘one-size-fits-all' model, particularly in elite sports. However, practitioners need guidance on the modalities of exercise prescription in challenging environmental conditions. The studies presented in the current Research Topic contribute to the translation of new findings into advices and actions. Bridging this gap requires a continuous effort based on *in-situ* athlete- and patient-centered studies, which we expect to have stimulated through this Research Topic. We hope this paves the way for further research in environmental challenges in sport and exercise sciences.

## Author contributions

All authors listed have made a substantial, direct, and intellectual contribution to the work and approved it for publication.

## Conflict of interest

The authors declare that the research was conducted in the absence of any commercial or financial relationships that could be construed as a potential conflict of interest.

## Publisher's note

All claims expressed in this article are solely those of the authors and do not necessarily represent those of their affiliated organizations, or those of the publisher, the editors and the reviewers. Any product that may be evaluated in this article, or claim that may be made by its manufacturer, is not guaranteed or endorsed by the publisher.
